# A novel ensemble approach for estimating the competency of bank telemarketing

**DOI:** 10.1038/s41598-023-47177-7

**Published:** 2023-11-27

**Authors:** Wei Guo, Yao Yao, Lihua Liu, Tong Shen

**Affiliations:** College of Innovation & Entrepreneurship, Shanghai Jianqiao University, Shanghai, 201306 Shanghai China

**Keywords:** Engineering, Mathematics and computing

## Abstract

Having a reliable understanding of bank telemarketing performance is of great importance in the modern world of economy. Recently, machine learning models have obtained high attention for this purpose. In order to introduce and evaluate cutting-edge models, this study develops sophisticated hybrid models for estimating the success rate of bank telemarketing. A large free dataset is used which lists the clients’ information of a Portuguese bank. The data are analyzed by four artificial neural networks (ANNs) trained by metaheuristic algorithms, namely electromagnetic field optimization (EFO), future search algorithm (FSA), harmony search algorithm (HSA), and social ski-driver (SSD). The models predict the subscription of clients for a long-term deposit by evaluating nineteen conditioning parameters. The results first indicated the high potential of all four models in analyzing and predicting the subscription pattern, thereby, revealing the competency of neuro-metaheuristic hybrids. However, comparatively speaking, the EFO yielded the most reliable approximation with an area under the curve (AUC) around 0.80. FSA-ANN emerged as the second-accurate model followed by the SSD and HSA with respective AUCs of 0.7714, 0.7663, and 0.7160. Moreover, the superiority of the EFO-ANN is confirmed against several conventional models from the previous literature, and finally, it is introduced as an effective model to be practically used by banking institutions for predicting the likelihood of deposit subscriptions.

## Introduction

### Background

Today’s modern world has witnessed many sophisticated advancements aiming at facilitating human life^1–3^. In this way, scientists have used different approaches (e.g., empirical and analytical) to better deal with complex problems^4–6^. Depending on the intended domain, the suggested solutions may comprise a wide range of responses to prediction tasks^7^, optimization-based simulations^8^, and network analysis^9,10^. Financial and economic issues are among the problems that have been highly benefitted from these advancements^11–13^. Assessing financial capacities^14^, risk evaluation of systems^15^, human resource management^16^, bank telemarketing^17^, etc. are some examples of the most notable applications in this sense.

As is known, telemarketing is defined as a system (or technique) for direct marketing in which the client is contacted in different ways to avail or purchase a product or facility^18^. The specific case of this action for banking subjects is called bank telemarketing^19^. Like many other financial parameters, having a reliable estimation of bank telemarketing would greatly assist institutions to develop and manage their plans toward a more sustainable system^20^. In this sense, many studies have focused on developing predictive models for predicting the success rate of bank telemarketing^21^.

### Literature review

Artificial intelligence models are among the new and convenient techniques that are developed for various purposes in the medical, engineering, financial sectors, etc.^22–24^. These methodologies all are based on mapping the dependence of a target parameter on several influential factors. In this way, a model may take advantage of non-linear algorithms for predicting the intended parameter. There are different kinds of machine learning tools that have properly served for predicting economic parameters and tasks related to banking like bankruptcy^25^, customer credit^26^, stock market^27^, credit rating^28^, etc.

Machine learning techniques have also received proper attention for predicting the success of telemarketing. Islam et al.^29^ used a naïve bayes (NB) algorithm to analyze for estimating the successfulness of bank telemarketing in which the influential parameters (i.e., features) were reduced from 150 to 17. Moro et al.^30^ applied an ANN for analyzing the customer lifetime value. They found the used methodology is suitable for improving the prediction performance only by taking the previous data. For instance, the area under the curve (AUC) of prediction increased from 0.8002 to 0.8609. In another effort, Moro et al.^31^ could successfully predict the success rate of telemarketing calls conducted to sell long-term deposits. Comparing four methodologies, namely logistic regression, ANN, decision trees, and support vector machine (SVM) showed that ANN presents the most reliable prediction. Utilizing a deep convolutional ANN, Kim et al.^32^ achieved 71% accuracy of prediction for the same purpose (i.e., predicting the success rate of telemarketing calls). Likewise, Desai and Khairnar^18^ used a hybrid of ANN and extreme gradient boosting applied to the telemarketing of a Portuguese bank that was affected by an economic crisis. The suggested model outperformed different typical machine learning models. Farooqi and Iqbal^33^ conducted a comparative study among several machine learning models such as NB, ANN, sequential minimal optimization, and k-nearest neighbor. With an accuracy of around 91.2%, the J48 decision tree outperformed other models.

Metaheuristic algorithms have been more recently introduced to have optimization roles in many fields of research. A broadly regarded application of these algorithms is being combined with regular machine learning models for optimizing their performance in estimating different parameters^34,35^. Not surprisingly, banking systems and financial sectors have widely benefitted from the use of optimization algorithms. For instance, Giri et al.^36^ showed the competency of biogeography-based optimization (BBO) for evaluating credit risk. Differential evolution (DE) used by Serrano-Silva et al.^37^ performed an automatic feature analysis for enhancing decision support systems in financial parts. Ajay Ram and Lakshmanarao^38^ applied a genetic algorithm (GA) to optimize the inputs of machine learning models (logistic regression, K-NN, random forest, decision tree, extra-tree classification, gradient boosting, AdaBoost classifier) in analyzing two datasets dealing with the prediction of (i) bank marketing performance and (ii) default of credit card clients. They achieved promising results and according to the comparisons adaboost classifier showed a superior performance for both datasets. Whale algorithms optimization (WOA) is another well-known metaheuristic technique that was employed by Yan et al.^39^ to optimize the parameters of a S_Kohonen network. The proposed algorithm could outperform GA.

### Innovation and contribution

The successful application of machine learning and metaheuristic algorithms in the financial sector indicates their efficiency in dealing with economic events and related parameters. Hence, it is expected to further employ these algorithms towards achieving a more sustainable economy and reducing the financial damages in banking institutions. Despite the wide application of conventional machine learning models^40,41^, the current literature lacks studies that highlight the applicability of metaheuristic-based hybrid models in these domains, particularly for predicting the competency of bank telemarketing. The authors are thereby encouraged to assess the suitability of four powerful metaheuristic techniques, namely electromagnetic field optimization (EFO)^42^, future search algorithm (FSA)^43^, harmony search algorithm (HSA)^44^, and social ski-driver (SSD)^45^ for predicting the subscription status of bank customers. In this way, these algorithms train an ANN to enable it to predict the subscription status of clients for a long-term deposit through bank telemarketing. The models are assessed in different ways and the best one is comparatively recognized. Moreover, the outstanding model of this study is compared to some conventional methods in the previous literature in order to highlight the efficiency of used metaheuristic algorithms and their solution for optimizing the weights and biases of the ANN model.

## Dataset description

Using proper data is of great importance in machine learning applications. An m-input n-target dataset represents how *n* target variables are affected by *m* influential parameters. The role of the data mining model, as the name indicates, is to discover this relationship. For the present research, a dataset which was introduced by Moro et al.^31^ is used. It is publicly available at https://archive.ics.uci.edu/ml/datasets/bank+marketing.

The target parameter here is a binary subscription status (BSS) of clients for a long-term deposit. More clearly, if the client subscribes, the response is Yes, otherwise No, noting that these conditions are quantified by 1 and 0 to be readable by the algorithm. This factor is considered to be influenced by 19 input factors, namely age, job, marital status, education, default credit status, housing, loan records, contact communication type, last contact time (month and day), number of contacts (before and during the campaign), the outcome of the previous campaign, the rate of employment variation, consumer price index, consumer confidence index, Euribor 3 months rate, and number of employees. Owing to the large number of parameters and their possible values/variables, the readers are kindly invited to refer to the reference paper^31^ and relevant webpage for the full details.

In the abovementioned webpage, several versions of this dataset can be found. Although the original dataset contains 45,211 records, the more suitable version includes 30,488 samples that are obtained after removing missing records/unknown labels. This version has been preferred in many previous studies^46,47^, and therefore, it has been used for the current research, too. Due to the objective of the research (purely assessing the performance of novel methods), the dataset was used without any preprocessing such as range normalization.

Next, 30,488 samples are randomly split into two different parts used for training the model and testing its generalizability. The first group is the larger one which contains 24,390 records, and the second group is formed by the remaining 6098 records. Random splitting is a common method for machine learning applications as it allows both training and testing datasets to have representatives from throughout the original large dataset. This task was done by “randperm” command in MATLAB.

## Methodology

### ANN

Generally, the ANNs are designed based on the actions taken place in a human neural system. The neurons which are the principal computational units are extremely connected to conduct the calculations toward obtaining the final output^48^. In an MLP, the structured layer and utilizing potent activation functions make it a universally known approximator for almost all complex parameters^49,50^. The neurons are placed in three subsequent layers, namely the input layer which is responsible for receiving the main data, the hidden layer (s) which performs the main calculations and non-linear analysis, and the output layer which releases the global output.

The process for calculating the output can be described in a general equation format as given in Eqs. (1) and (2):

1 $$Sum =Input \times weight+bias$$

2 $$Output =f(Sum)$$

where *f()* is the activation function executed by the neurons.

This operation is first carried out by hidden units, and they yield the outcome value to the output unit. It is then again performed using new weights and biases to let the network predict the global response.

### Metaheuristic algorithms

The metaheuristic algorithms used in this study play the role of trainers for the MLP. Through an optimization process, it minimizes the training errors by trying a large number of configurations for a predefined ANN. Each algorithm has complicated specifications for performing the optimization. Due to the large number of models used in this study, the algorithms are briefly introduced in this section and appropriate references are presented for further mathematical details.

The EFO, as its name connotes, is a simulation of electromagnetic fields based on attraction–repulsion regulations. It presents a fast and reliable metaheuristic optimizer developed by Abedinpourshotorban et al.^42^. The optimization using EFO happens for updating the electromagnet particles which are grouped in positive, negative, and neutral fields. The EFO is expressed with computation detailed in previous literature^51,52^.

The mechanism of the FSA algorithm, developed by Elsisi^43^, is an imitative effort of what people do to improve their life quality. The candidate solutions are considered as local solutions and the best one among them is the global solution. A significant property of this algorithm is that, unlike others, the irregular population is refreshed in each repetition. The location of the population is updated and the solution is improved. This technique is mathematically described in earlier studies^53,54^.

Geem et al.^44^ designed the HSA algorithm whose main idea is changing the pitches of an instrument. The objective is to create new harmonies. Similar to other population-based techniques, first the parameters are set and the population is scattered. Next, the fitness of each solution is evaluated and the worst one is replaced with a new promising solution. This process continues until a stopping criterion is met. For a more detailed formulation of the HSA, the readers may refer to previous works^55,56^.

Tharwat and Gabel^45^ proposed the SSD algorithm. Basically, the SSD is a simulation of a downward path of ski-drivers which considers the agents’ position and the candidate solution. The SSD uses the mechanism of different optimizers. For instance, it mimics the PSO algorithm for updating the solution and comparing it with future ones and mimics the GWO algorithm for finding a mean global solution. This algorithm is extensively formulated in earlier research^57,58^.

### Accuracy criteria

Three accuracy criteria are employed in this study to evaluate and rank the performance of the predictive models. Two famous error indicators are root mean square error (RMSE) and mean absolute error (MAE). The formulations of the RMSE and MAE are expressed in Eqs. (3) and (4), respectively.

3 $$RMSE=\sqrt{\frac{1}{K}\sum_{i=1}^{K}{({BSS}_{{i}_{observed} }-{BSS}_{{i}_{predicted} })}^{2}}$$

4 $$MAE= \frac{1}{K}\sum_{i=1}^{K}\left|{BSS}_{{i}_{observed} }-{BSS}_{{i}_{predicted} }\right|$$

where ${BSS}_{{i}_{observed}}$ and ${BSS}_{{i}_{predicted}}$ stand for the observed and predicted BSSs pertaining to the *i*^*th*^ pair out of *K* pairs,

Moreover, the area under the curve (AUC) index which measures the area under the receiver operating characteristic (ROC) curve, is considered to report the accuracy of prediction based on Eq. (5).

5 $$AUC = \frac{\sum TP+\sum TN}{(P + N)}$$

where P and N are the total number of BSSs with Yes and No responses, respectively. Also, FP, TP, FN, and TN represent false positive, true positive, false negative, and true negative respectively.

## Results and discussion

The results of this study are mainly the prediction potential of the suggested models. To assess it, the models first need to be trained. The quality of training shows how well the model can learn the BSS pattern, while the prediction ability is determined by assessing the testing data.

When a metaheuristic algorithm is involved, the first step for training is structuring the ANN as the skeleton. The algorithm then is applied to this row skeleton to make it learn and predict. In this research, the number of inputs and outputs was 19 and 1, respectively, which dictates an ANN with 19 and 1 neurons in the input and output layer. The number of neurons in the middle layer was set to 8 after trying different configurations. With the same trial and error logic, the activation functions of the hidden and output layers are selected to be Tansig and Purelin, respectively. Therefore, the final configuration of the ANN is MLP (19, 8, 1). In the next step, it is explained how a metaheuristic algorithm trains the specified ANN.

### Training results

**Figure 1:**
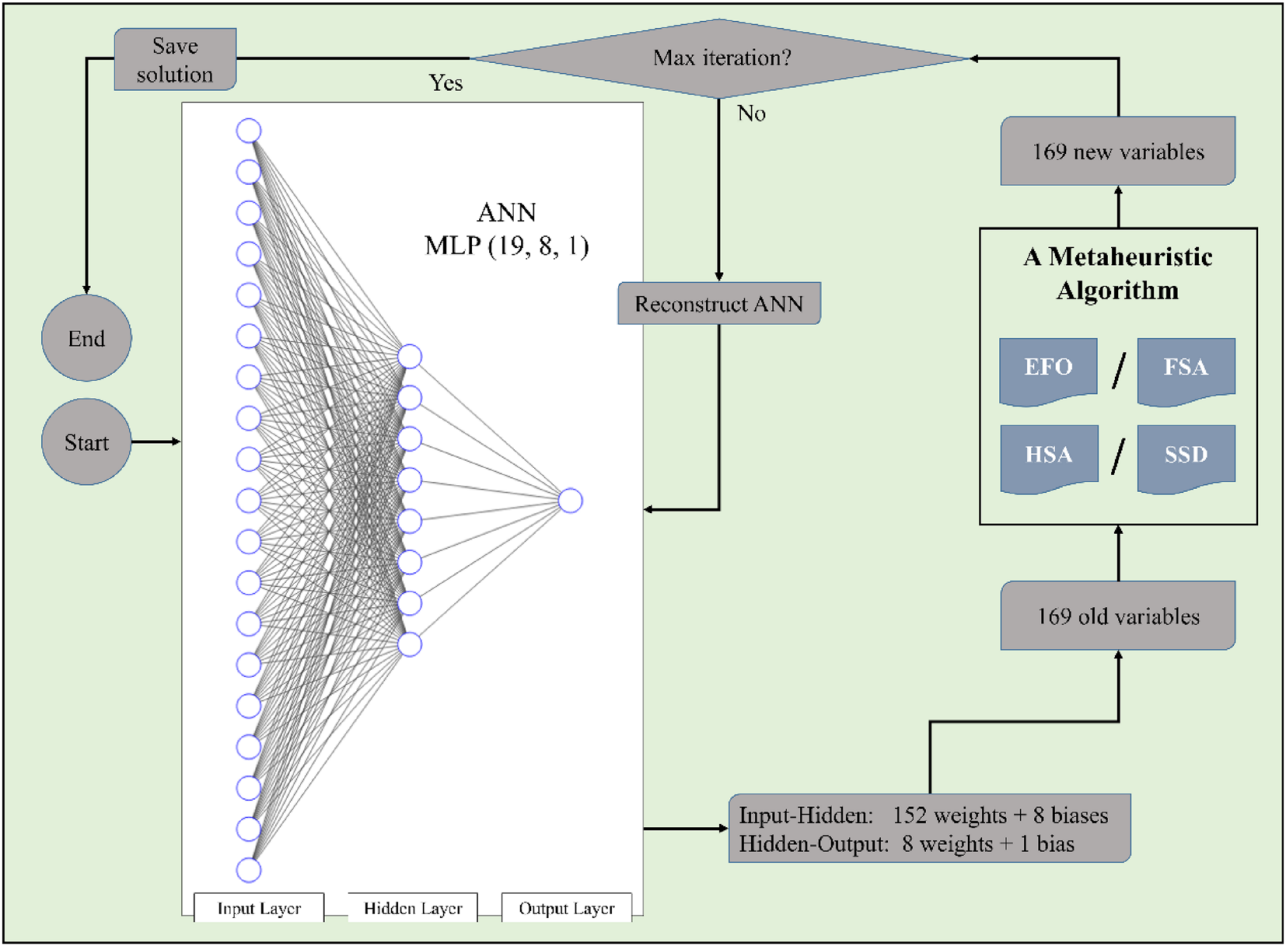
Schematic view of the optimization process of ANN using metaheuristic algorithms.

Combining the models is the first step of the optimization task. In this sense, the ANN is exposed separately to the EFO, FSA, HSA, and SSD algorithms to create the EFO-ANN, FSA-ANN, HSA-ANN, and SSD-ANN hybrids. In these configurations, the ANN is optimally trained by repetitive efforts. Figure 1 depicts the optimization process. In each iteration, a total of 169 optimized weights and biases are suggested for constructing the ANN.

Two important parameters of each metaheuristic algorithm are the number of repetitions and the population size. Based on an extensive trial and error effort, the best performance of the EFO, FSA, HSA, and SSD was found with population sizes of 40, 400,400, and 400, respectively. The number of repetitions is determined based on the behavior algorithm. For instance, in this work, the EFO performs 50,000 repetitions, while the FSA, HAS, and SSD are good with 1000. In each repetition, the result is represented by a value called objective function which applies Eq. (3) to the training samples, i.e., training RMSE. Since it is set to be the RMSE criterion here, the lower the objective function comes, the more accurate the training is.

**Figure 2:**
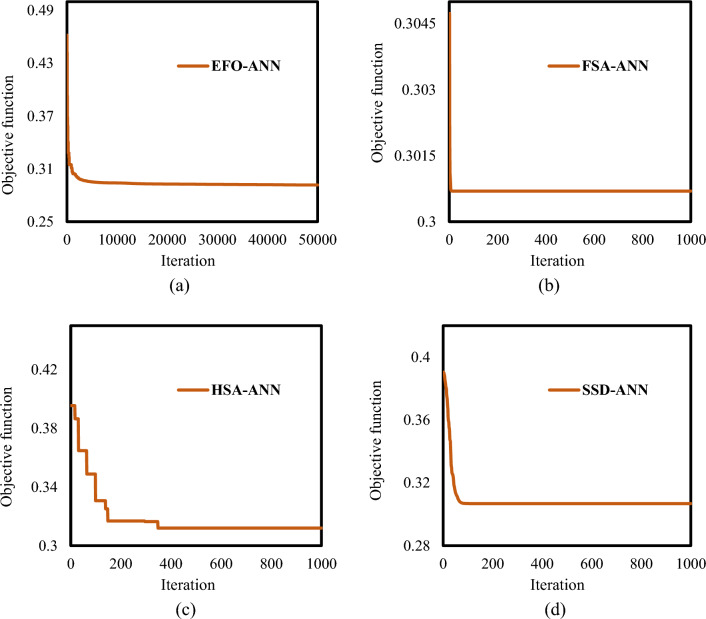
Optimization curves of (**a**) EFO-ANN, (**b**) FSA-ANN, (**c**) HSA-ANN, and (**d**) SSD-ANN.

Figure 2 shows the optimization results. In these figures, each algorithm has a different behavior for optimizing the ANN. For instance, the HSA has a stepwise reduction path, while the other three have a sharp fall in the beginning. Finally, the objective function values are 0.2917, 0.3007, 0.3120, and 0.3068 for the EFO-ANN, FSA-ANN, HSA-ANN, and SSD-ANN.

**Figure 3:**
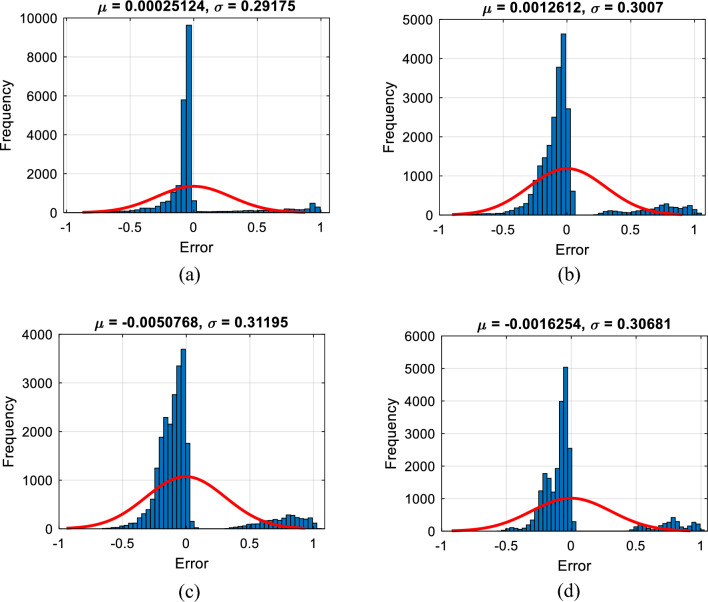
Histogram of errors of (**a**) EFO-ANN, (**b**) FSA-ANN, (**c**) HSA-ANN, and (**d**) SSD-ANN.

Figure 3 shows the histogram of the training records. As is known, the higher the frequency of errors around zero, the more accurate the results. From these figures, the distribution of error values is fine and indicates the majority of the BSS values have been accurately predicted by the models. In this sense, the MAE values were 0.1706, 0.1840, 0.1994, and 0.1945.

The third accuracy criterion is AUC whose values were 0.8030, 0.7809, 0.7356, and 0.7634. These values all indicate an acceptable range of accuracy for all models, especially, the EFO-ANN whose accuracy is above 80%.

### Testing performance

**Figure 4:**
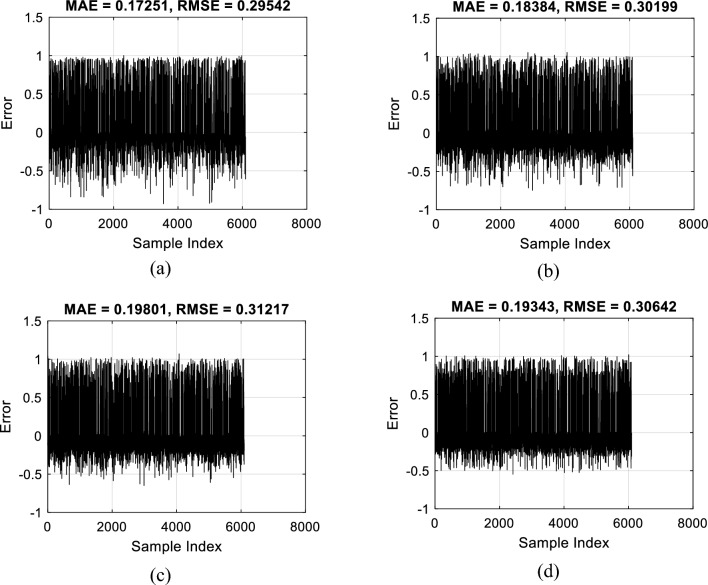
Testing error analysis of (**a**) EFO-ANN, (**b**) FSA-ANN, (**c**) HSA-ANN, and (**d**) SSD-ANN.

Once the training is completed, testing data are exposed to the acquired knowledge of the models to see how accurate they can predict the BBS for unseen client conditions. Figure 4 illustrates the error of prediction for each pair. The RMSE values of 0.2954, 0.3020, 0.3122, and 0.3064 represent a satisfying accuracy in this step. Moreover, the MAEs were 0.1725, 0.1838, 0.1980, and 0.1934.

**Figure 5:**
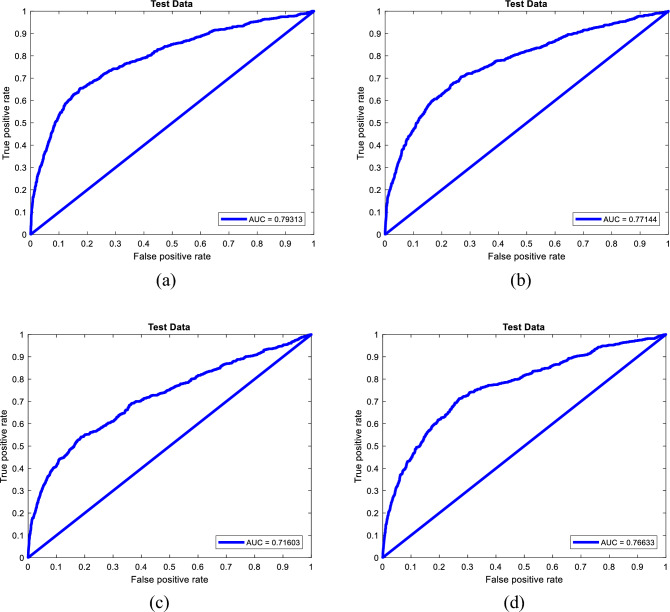
The ROC diagrams for testing performance of (**a**) EFO-ANN, (**b**) FSA-ANN, (**c**) HSA-ANN, and (**d**) SSD-ANN.

Similar to the training phase, the above assessments are followed by the AUC evaluation. Figure 5 shows the ROC curves belonging to the testing phase of each model. The areas under these curves are 0.7931, 0.7714, 0.7160, and 0.7663, indicating a high level of accuracy.

It was previously explained that the target values are 1 and 0. The testing products of the EFO-ANN, FSA-ANN, HSA-ANN, and SSD-ANN fell within [− 0.1118, 0.9648], [− 0.0604, 0.7724], [− 0.0840, 0.6807], and [− 0.0355, 0.5748], respectively. To evaluate the rate of correctly classified BSSs, the predicted values higher than 0.5 and below 0.5 are rounded into 1 and 0, respectively. It showed that while the HSA-ANN classified 88.22% correctly, the other three models achieved 88.76% accuracy. But in the training phase, the difference was more sensible. This value for the EFO-ANN, FSA-ANN, HSA-ANN, and SSD-ANN models was 89.03, 88.67, 87.82, and 87.15, respectively.

Three accuracy indices together, all used models could achieve a reliable prediction for the BSS. In other words, when the models are properly trained using previous records, they can be used to predict future conditions with high accuracy. Hence, the models are verified to be used for practical purposes in financial institutions.

### Comparison

Although suitable accuracy levels were provided by all models, the primary aim of this research has been to compare the models and introduce the most reliable ones. In this sense, the accuracy of the models is compared in this section. Table 1 collects these values.

**Table 1:** Summarized accuracy assessment results.

Phase	Model	MAE	RMSE	AUC	Classification accuracy (%)
Train	EFO-ANN	0.1706	0.2917	0.8030	89.03
	FSA-ANN	0.1840	0.3007	0.7809	88.67
	HSA-ANN	0.1994	0.3120	0.7356	87.82
	SSD-ANN	0.1945	0.3068	0.7634	87.15
Test	EFO-ANN	0.1725	0.2954	0.7931	88.76
	FSA-ANN	0.1838	0.3020	0.7714	88.76
	HSA-ANN	0.1980	0.3122	0.7160	88.22
	SSD-ANN	0.1934	0.3064	0.7663	88.76

In the training phase, all accuracy criteria reflect the superiority of the EFO-ANN, followed by the FSA-ANN, SSD-ANN, and HSA-ANN. The EFO-ANN achieved the lowest RMSE and MAE, as well as the greatest AUC. This comparison reveals that the EFO can optimize the ANN by providing proper weights and biases for a predefined neural network.

As for the test phase, the same ranking is observed for all models. The EFO-ANN reached the highest level of accuracy. There were meaningful distinctions between the AUCs of the models (i.e., 0.7931 vs. 0.7160). In this regard, the HSA-ANN presented the poorest prediction of the BSS.

Considering the structure of the used ANN, i.e., 19 × 8 × 1, there were a total of 169 variables to be adjusted during each optimization. This task was considerably better done by the EFO algorithm, and the suggested variables built a network that could achieve the best reproduction of the BSS. The EFO also could perform the optimization task with a considerably less complicated configuration. It used a population size that was one-tenth of other algorithms (40 vs. 400, see Section “Training results”). Considering the time of optimization, the EFO took around 724 s, while this time equals 29,251, 3480, and 10,562 for the FSA, HAS, and SSD, respectively.

In comparison with previous literature, the EFO-ANN of this study achieved significant improvements. It classified 88.76% of the samples correctly and achieved an AUC of 79.31% which is higher than many conventional methods. For instance, Camacho-Urriolagoitia et al.^59^ applied various machine learning tools to financial problems. As far as the problem of this study is concerned, six models of Naïve Bayes, Logistic, kNN, SVM, MLP, and AdaBoost could correctly classify 76.80%, 85.76%, 86.25%, 76.63%, 88.01%, and 86.58% of the samples, respectively (see “Bank Additional” results in Table 3 of the cited study). In comparison with 88.76% which is obtained in this work, it can be said the EFO-ANN shows comparably higher promise. Furthermore, by comparing the results of EFO-ANN (i.e., EFO-MLP) in this study and classical MLP in Ref.^59^, the effectiveness of the EFO algorithm in enhancing the MLP model is deduced. In another example, a deep convolutional neural network used by Kim et al.^32^ achieved 76.70% accuracy and it outperformed several other conventional methods. These results are below 79.31% accuracy obtained by the EFO-ANN of this study. Similar superiority can be derived for the EFO-ANN versus several extreme learning machine and deep learning models tested by Koçoğlu and Esnaf^60^ (see Tables 4 and 5 of the cited study). Likewise, the overall accuracy of two models, namely SVM and S_Kohonen network employed by Yan et al.^39^ is lower than the suggested EFO-ANN (see Fig. 2 of the cited study).

### Further discussion and future efforts

While the used models achieved an acceptable level of accuracy, this research had some limitations that addressing them can increase the clarity of the work. For instance, since the primary aim of the study was concerned with introducing novel methods for BSS prediction, both the dataset and implementation strategies were in the original form. More clearly, the authors believe that there are some ideas for improving the use of the dataset and implementing the models that may result in a better accuracy of prediction.

The below suggestions can be regarded in future studies for enhancing the validation/applicability of the results:

- Conducting a pre-processing in order to eliminate outliers and normalization.
- Feature analysis and importance assessment to see which parameters have a greater contribution to BSS, thereby, creating an input-reduced dataset. It helps with reducing the dimension of the problem, and therefore, more efficient optimization.
- Enriching the existing dataset with the records of external datasets (e.g., from countries other than Portugal) to possibly broaden the generalizability of the models.
- Validating the predictive performance of the models using external datasets.
- Performing k-fold cross-validation instead of random division to attain a more reliable accuracy.
- Replacing the ANN with other machine learning models such as adaptive neuro-fuzzy inference system (ANFIS) to evaluate their accuracy.
- Including more recent metaheuristic algorithms and conducting comparative efforts in order to keep the solution updated with the latest developments.
- Extracting a mathematical formula from ANN-based models that may serve for a more convenient prediction of BSS.

## Conclusions

In order to find a reliable predictive model for BSS modeling, a series of new metaheuristic algorithms were comparatively evaluated in this research. Electromagnetic field optimization, future search algorithm, harmony search algorithm, and social ski-driver algorithms were assigned to the training of an ANN. Based on the training results, all models could capture the behavior of BSS from the historical data of a Portuguese bank with high accuracy. Concerning the prediction ability, all models could nicely forecast the behavior of customers in terms of subscribing for a long-term deposit. Above all, the EFO-ANN was the superior hybrid model based on all accuracy indicators. This model could also outperform several conventional techniques used in the previous literature. Thus, it can be an efficient suggestion for financial institutions to predict the behavior of their clients in order to avoid risk. For future works, similar metaheuristic algorithms can be used in combination with other basic predictors for computational improvements. Also, exposing the data of different banking institutions and strong data preprocessing are highly recommended for developing a more generalizable solution.

## Data Availability

The source of all data analyzed during this study is freely accessible as introduced in Section “Dataset description” of this article.
