# Author Correction: A novel ensemble approach for estimating the competency of bank telemarketing

**DOI:** 10.1038/s41598-024-51673-9

**Published:** 2024-01-11

**Authors:** Wei Guo, Yao Yao, Lihua Liu, Tong Shen

**Affiliations:** College of Innovation & Entrepreneurship, Shanghai Jianqiao University, Shanghai, 201306 Shanghai China

Correction to: *Scientific Reports* 10.1038/s41598-023-47177-7, published online 27 November 2023

The original version of this Article contained an error in the spelling of the author Wei Guo which was incorrectly given as Gei Guo.

The original Article has been corrected.

